# Acute lower limb ischemia and intestinal necrosis due to arterial tumor embolism from advanced lung cancer: a case report and literature review

**DOI:** 10.1186/s40792-018-0452-1

**Published:** 2018-05-02

**Authors:** Sayuri Togo, Terutoshi Yamaoka, Kazutoyo Morita, Kazuomi Iwasa, Yukihiko Aoyagi, Yumi Oshiro, Takatoshi Fujishita, Hideki Yokoyama, Takashi Matsui, Takashi Nishizaki

**Affiliations:** 1 Department of Surgery, Matsuyama Red Cross Hospital, 1, Bunkyo-cho, Matsuyama-shi, Ehime 790-8524 Japan; 2 Department of Vascular Surgery, Matsuyama Red Cross Hospital, Matsuyama-shi, Ehime Japan; 3 Department of Pathology, Matsuyama Red Cross Hospital, Matsuyama-shi, Ehime Japan; 4 Departments of Thoracic Surgery, Matsuyama Red Cross Hospital, Matsuyama-shi, Ehime Japan

**Keywords:** Arterial tumor embolism, Lung cancer, Acute mesenteric ischemia, Acute limb ischemia

## Abstract

**Background:**

Arterial tumor embolism (ATE) is a rare but life-threating complication.

**Presentation of case:**

A 55-year-old man with acute lower-limb ischemia was referred to our hospital after endovascular intervention failed and underwent above-the-knee amputation for severe limb necrosis. On postoperative day 8, he developed small bowel necrosis and underwent resection. Histopathological examination of the resected bowel revealed that the submucosal arterial emboli were positive for the markers of squamous cells. He had unresectable lung squamous cell carcinoma with left atrium invasion. The subsequent embolisms were thought to be caused by the advanced lung cancer.

**Conclusion:**

ATE is rare but should be considered as a differential diagnosis for unidentified arterial occlusion.

## Background

Arterial tumor embolism (ATE) is often a fatal and rare complication of tumors. We herein report a case of ATE that spontaneously occurred after chemoradiotherapy of primary lung cancer. The embolus eroded through the wall of a pulmonary vein and embolized through fragmentation with resultant showering of tumor emboli to multiple arterial sites.

## Case presentation

**Fig. 1:**
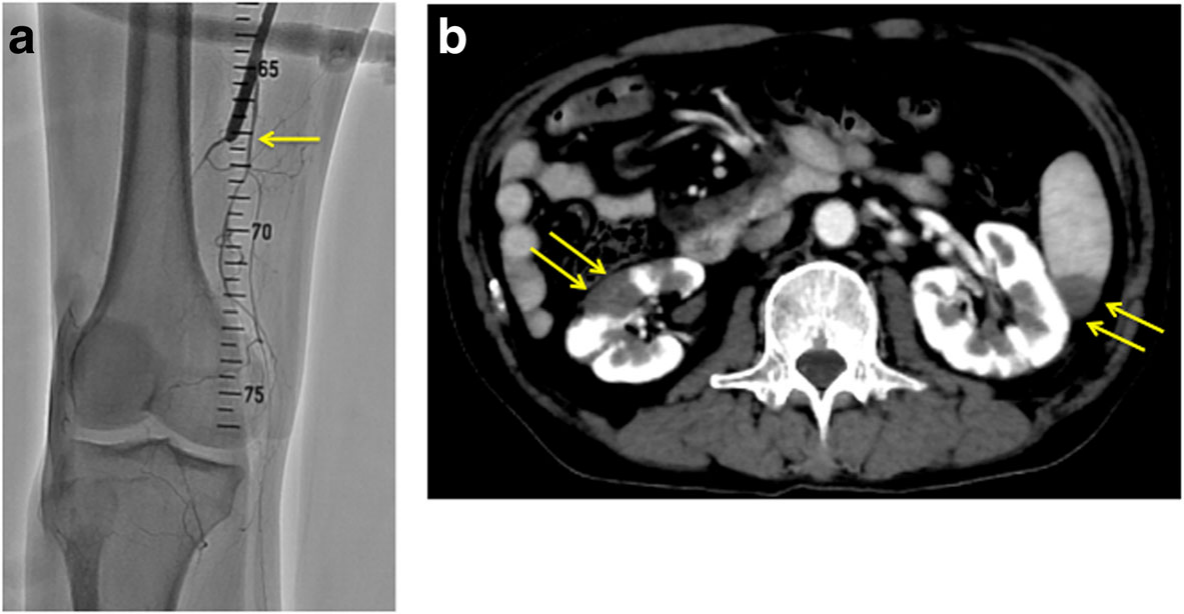
**a** Angiography revealed obstruction of right distal superficial femoral artery. **b** Abdominal contrast-enhanced CT scan showed partial infarction of right kidney and spleen

A 55-year-old man presented to a local hospital, complaining of acute severe pain in his right lower leg. He was diagnosed with acute lower limb arterial embolism and underwent endovascular intervention: percutaneous aspiration of thrombus and balloon angioplasty of right distal superficial femoral artery (SFA) (Fig. 1a). After intervention, he began taking of clopidogrel and acetylsalicylic acid and heparinisation was started. The next day, he developed reocclusion of distal SFA and was transferred to our hospital.

When he was transferred to our hospital, his right lower limb showed completely irreversible ischemic changes: pallorous and cold skin, loss of sensation, muscular rigidity of the right ankle, non-dopplerable right dorsalis pedis, and posterior tibial pulses with only the right femoral pulse above the inguinal ligament palpable. He had no medical history of chronic peripheral arterial occlusive diseases. He had well-controlled hypertension and diabetes mellitus. Eight months prior, he had been treated with chemoradiotherapy in another hospital for unresectable lung cancer: squamous cell carcinoma, located in the right median and inferior lobes of the lung.

His white blood cell count was 21,350/μl. Other blood chemistry values were serum high-sensitivity C-reactive protein: 24.93 mg/dl; creatinine: 2.49 mg/dl; creatinine kinase: 18,375 U/l; myoglobin: 34,155 ng/ml; and PT-INR: 1.00; APTT 32.4 s. Abdominal contrast-enhanced computed tomography (CT) scan showed partial infarction of the spleen and right kidney (Fig. 1b) and no aortic disease which would develop embolism. The electrocardiogram showed sinus rhythm. We saw no findings of thrombus, vascular disease, or shunt in the echocardiogram. Since limb salvage was impossible, above-the-knee amputation was performed. Based on these clinical findings, we diagnosed acute lower limb ischemia due to emboli, probably of cardiac origin; he was postoperatively treated with an anticoagulant (rivaroxaban).

**Fig. 2:**
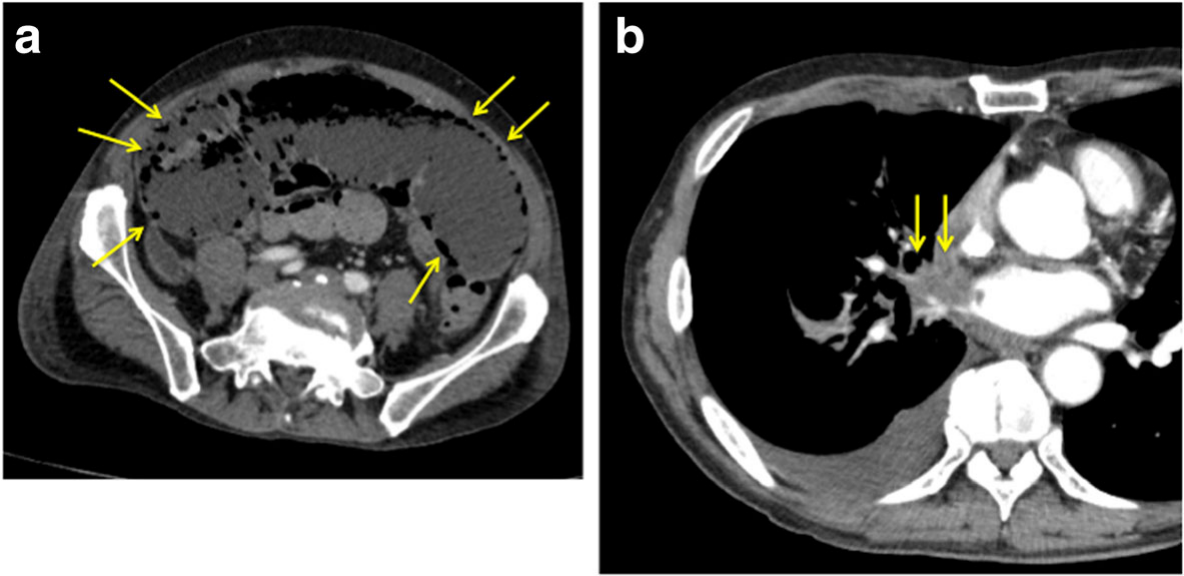
**a** Abdominal contrast-enhanced CT scan showed diffuse intestinal and mesenteric emphysema. **b** A postoperative CT scan showed a lobulated 6.5-cm tumor in the right lung had directly invaded the inferior pulmonary vein and left atrium

On postoperative day (POD) 1, he started his meal. However, on POD5, he became unable to eat because of abdominal distention. On POD8, the patient developed severe, constant abdominal pain. Abdominal contrast-enhanced CT scan showed diffuse intestinal and mesenteric emphysema (Fig. 2a). A small branch of superior mesenteric artery has partially embolised, but the distal vessel had good blood flow. We saw no obvious mesenteric arterial occlusion. He underwent emergency laparotomy for small bowel necrosis. The small bowel was segmentally and diffusely necrotic and was perforated at the proximal jejunum (30 cm from Treitz ligament). However, the mesenteric arterial pulsations were visible and palpable throughout the small intestine. The proximal jejunum 20 cm from Treitz ligament and distal ileum 60 cm from terminal ileum were intact. We resected approximately 400 cm of necrotic small bowel. The remnant small bowel was anastomosed.

**Fig. 3:**
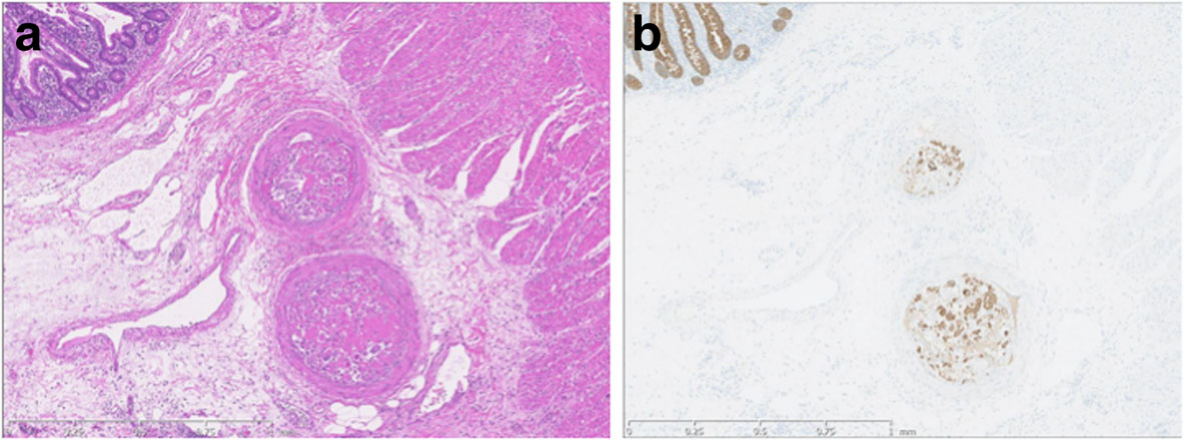
**a** Histopathological examination revealed submucosal arterial thrombosis including denatured cells (hematoxylin and eosin; × 25). **b** Immunohistochemically, submucosal arterial thrombosis was positive for AE1/AE3 (cytokeratin; AE1/AE3; × 25)

Histopathological examination of the resected specimen revealed ischemic changes and submucosal arterial thrombosis, including denatured cells (Fig. 3a). Immunohistochemically, they were positive for cytoketatins, AE1/AE3, 34βE12, and CK5/6 (Fig. 3b). A postoperative CT scan showed that a lobulated 6.5-cm tumor in the right lung had directly invaded the inferior pulmonary vein and left atrium (Fig. 2b). In retrospect, X-ray examination of the chest on admission showed a similar finding. Subsequent embolism was thought to be caused by the advanced lung cancer.

He was withdrawn from central-vein nutrition with no complications on POD 22 after bowel resection. After the care and additional amputation of the femoral stump, he was transferred to a rehabilitation hospital in good condition on POD 84 after his initial amputation, continuing taking rivaroxaban according to the treatment of chronic atrial fibrillation.

## Discussion

Acute arterial occlusion secondary to malignant tumor embolism is rare. Miroslav et al. reviewed 877 cases of arterial emboli, of which only three cases (0.3%) of ATE were identified [1]. Xiromeritis et al. reported that the majority (89.4%) of ATE were associated with primary lung cancer (44.2%), secondary lung cancer (31.7%), and primary tumors of the aorta (13.4%) [2]. To our knowledge, only four cases of primary lung cancer that embolized to the superior mesenteric artery have been reported besides the present case (Table 1).

**Table 1:** Published reports of primary lung cancer that embolised to the superior mesenteric artery

	Primary lung tumor	Pulmonary vein involvement	Site of embolization	Treatment	Outcome
Taber [8]	Anaplastic carcinoma	Y	Distal aorta, SMA, cerebral and renal arteries	Embolectomy	Died (postoperative hour 15)
Webb [9]	Bronchogenic carcinoma	Y	Distal aorta, cerebral artery, and SMA	Embolectomy, laparotomy	Survived
Whyte [10]	Large cell carcinoma	Y	SMA	Laparotomy	Died (POD4)
Chandler [4]	Squamous cell carcinoma	Not noted	SMA	Laparotomy	Died (POD8)
Togo (this article)	Squamous cell carcinoma	Y	Renal, splenic and femoral arteries, the branch of SMA	Laparotomy, trans-femoral amputation	Survived

*SMA* superior mesenteric artery, *Y* yes

The prognosis of ATE is very poor and embolectomy should be attempted for recovery. The longest recorded survival after tumor embolectomy was 20 months [3]. In that paper, Richard et al. reported that among patients who survived embolectomy, the presence of tumor embolism did not affect the prognosis. It was highly correlated with the TNM staging of the primary lung tumor. However, most patients with cerebral and cardiac ATE could not undergo embolectomy, and their mortality approached 100% [4].

Factors that predispose a patient to ATE are size, rapidity of tumor growth, number, survival rate of malignant cells, and poorly differentiated cancer [4]. Invasion into pulmonary veins and the left atrium is also undoubtedly important. Surgical manipulation of a tumor with invasion into the pulmonary veins is likely to be a risk factor for ATE [4, 5].

Treatment of ATE includes early heparinization to prevent propagation of distal thrombus, and embolectomy using a Fogarty catheter, or laparotomy and embolectomy with resection of the infarcted intestine and primary anastomosis, which is same as that for atherosclerotic emboli.

The patient in this case survived for three reasons. First, he did not have cerebral or cardiac ATE. Second, he underwent surgical treatment immediately. Finally, the remnant small bowel was not necrotic.

Organ infarction or ischemia is occasionally the presenting feature of a previously undiagnosed primary tumor [6]; ATE should be considered for patients with unidentified ischemia.

This patient had previously undergone chemotherapy with cisplatin + docetaxel (1st line), tegafur/gimeracil/oteracil (2nd line), nab-paclitaxel (3rd line), and nivolumab (4th line). Nivolmab was administered 8 months before admission. Nivolumab is reported to provide a durable response that persists after discontinuation. Tapalian et al. reported 12 (71%) of 17 patients maintained responses off-therapy for at least 16 weeks (range 16–56 weeks) [7]. The present patient’s primary lung tumor might have shrunk after the administration of Nivolmab.

## Conclusion

ATE is rare but should be considered as a differential diagnosis for unidentified arterial occlusion.

## Abbreviations

ATE: Arterial tumor embolism
CT: Computed tomography
POD: Postoperative day
SFA: Superficial femoral artery
